# Editorial: The Role of Mast Cells in Immediate Hypersensitivity Reactions

**DOI:** 10.3389/fimmu.2021.780829

**Published:** 2021-10-13

**Authors:** Marcelo Vivolo Aun, Natalia Blanca-López, Mariana C. Castells, Pedro Giavina-Bianchi

**Affiliations:** ^1^ Division of Host & Defense, Faculdade Israelita de Ciências da Saúde Albert Einstein School of Medicine, São Paulo, Brazil; ^2^ Clinical Immunology and Allergy Division, University of São Paulo School of Medicine, São Paulo, Brazil; ^3^ Allergy Service, Hospital Universitario Infanta Leonor, Madrid, Spain; ^4^ Division of Allergy and Clinical Immunology, Department of Medicine, Brigham and Women’s Hospital, Harvard Medical School, Boston, MA, United States

**Keywords:** mast cells, activation, urticaria, hypersensitivity, mastocytosis

Mast cells are important cells at the cross roads of innate and adaptive immunity. In an interesting review paper about mast cells functions, Krystel-Whittemote described that they are present in all organs systems and thought to play essential roles in the maintenance of many physiological functions as well as in the pathophysiology of many diseases. They originate from pluripotent progenitor cells in the bone marrow, and, under normal conditions, immature mast cell progenitors travel in the blood stream, migrate into peripheral tissues and differentiate into mature mast cells under the influence of stem cell factor and various homing cytokines (1). Typically, mature mast cells do not circulate in the bloodstream.

Allergic diseases are associated with mast cell activation, degranulation, and release of pre-formed mediators, leading to clinical manifestations such as urticaria, angioedema, bronchospasm and anaphylaxis. These symptoms can be induced by many triggers which can induce IgE-and non IgE-mediated reactions, and can occur as the result of an increased mast cell burden, as in mastocytosis (2).

In the current Research Topic of Frontiers in Immunology, articles describing recent advances in mast cell activation disorders are presented, which discuss new pathophysiological mechanisms and emerging therapeutic targets in the management of those conditions.

One important receptors involved in mast cell activation is the Mas-related G-protein-coupled receptor X2 (MRGPRX2), which has been linked to several mast cell-related diseases, such as chronic spontaneous urticaria, atopic dermatitis and asthma. This receptor is expressed by different mast cell subsets and it induces degranulation upon binding by different ligands such as quinolone antibiotics, general anesthetics such as atracuronium and rocuronium and positively charged, hydrophobic molecules such as vancomycin and morphine, as cited in a recent review manuscript by McNeil. (3, 4). Working on *in vitro* cells, Hermans et al. showed that HMC1 cells express the receptor MRGPRX2, at lower levels compared to LAD2 and HuMC cells and pre-incubating these cells with latrunculin-B leads to overexpression of MRGPRX2, which can be activated by compound 48/80, resulting in efficient HMC1 degranulation. Their findings suggest that HMC1 cells may be used to study mast cell activation through MRGPRX2. Oliveira et al. described that annexin A1, an endogenous 37 KDa glucocorticoid induced monomeric protein, which inhibits MC degranulation in murine models, is capable of interfering with the activation of HMC-1 cells. *In vivo* intraperitoneal administration of AnxA1to wild type and IL-4 knock-out mice reduced mast cell activation, suggesting its potential therapeutic use to reduce the release of MC mediators in inflammatory allergic processes.

Two further studies address new pathways of mast cells inhibition that could be investigated as potential future therapeutic targets. Li et al. describe the orosomucoid-like-3 (ORMDL3) gene, which regulates the endoplasmic reticulum stress (ERS)-induced unfolded protein response (UPR) and autophagy, and show that its protein product can suppresses Ag-mediated mast cell activation *via* an ATF6 UPR-autophagy dependent pathway, attenuating anaphylactic reactions.

In a murine model of active cutaneous anaphylaxis (ACA), Bonamichi-Santos et al. demonstrated that the programmed cell death ligand 1 (PDL-1), which is known for its inhibitory effect on T cell immune response and is expressed on the surface of mast cells, may have a relevant role in allergic diseases. Using a monoclonal antibody anti-PD-L1, the authors showed that PD-L1 blockade during allergen sensitization inhibited the synthesis of specific IgE and IgG1 and decreased mast cell activation. This effect was not observed when anti-PD-L1 was administered before antigen challenge, suggesting that the effect of blocking PD-L1 pathway affects the induction phase of the immune response not its effector phase.

Elst et al. provide further evidence of the functions of the MRGPRX2 using peripheral blood-derived cultured mast cells from healthy donors and drug allergic patients in order to assess mast cell activation and degranulation through MRGPRX2 and after silencing its effect. They show that atracurium, ciprofloxacin, and levofloxacin induced activation and degranulation in primary human mast cells, but only in MRGPRX2-positive and not in MRGPRX2-negative or -silenced mast cells. Sugammadex attenuated the atracurium-induced activation and degranulation of human mast cells through MRGPRX2 by reducing free atracurium levels.

Two different studies addressed cold urticaria and its association with the presence of cryoproteins, such as cryoglobulins and cold agglutinins. In the first article, Bizjak et al. investigated 35 cold urticaria patients and found that 46% of them had a positive cold agglutinin test, while 27% had a positive cryoglobulin test. They demonstrated that a positive cold agglutinin test, but not a positive cryoglobulin test, was associated with a higher rate of reactions triggered by cold weather and by exposure to cold water, aggravated by increased humidity. Patients with a positive cold agglutinin test had a higher frequency of angioedema triggered by ingestion of cold foods or drinks, and lower disease control. Ginter et al. looked for evidence of the association between cryoproteins and cold urticaria. They performed initially a systematic review and identified 14 studies including 1151 cold urticaria patients. The meta-analysis showed a low frequency of cryoproteins in those patients, from 0.7% for cold agglutinins to 3.0% of cryoglobulins. They then performed a retrospective analysis of 293 individuals in a single Center of Reference in a 5 years period and found low frequency of cryoproteins at 4.1%. Finally, they prospectively studied 49 cold urticaria individuals and found a very low frequency of cryoproteins: none with cryoglobulins, none with cryofibrinogens, and 2/46 (4.3%) with cryoagglutinins, who did not have underlying autoimmune or hematological disorders, indicating that the pathogenesis of cold urticaria is independent of cryoproteins.

In a large case series of more than 600 CSU patients, Sauer et al. investigated the relation between IgA and IgE levels and autoimmunity and autoreactivity. They found that lower IgA levels were associated with lower IgE levels, a higher frequency of recurrent angioedema, autoimmunity, and elevated levels of IgE-anti-thyroid peroxidase.

CSU patients have been successfully treated with second generation antihistamines, some of them with higher doses, but refractory CSU is very common in clinical practice. Omalizumab have been considered the first-line medication indicated for patients with CSU who do not respond to a four-fold dose of non-sedating antihistamines (5). Liao et al. described two pregnant women who presented with refractory CSU and were safely and successfully treated with omalizumab. They review the literature and found 11 pregnant women who were safely treated with omalizumab who had resolution of symptoms and successful deliveries.

Barni and colleagues reported a pediatric patient who developed refractory CSU associated with Chron’s Disease (CD) and who was safely and successfully treated with omalizumab. She started therapy for CD with azathioprine and mesalazine at the age of 12 without any disease relapse. When she was 17, she began to present wheals and angioedema, which were not controlled with high doses of antihistamines. Omalizumab was added-on to CSU therapy, with a good clinical response and no relapse in CD.

Last but not least the role of mast cell activation in patients with clonal mast cell disorders is provided by Rama et al. who reported an 18-year-old male who presented a long-standing history of atypical urticarial skin lesions, allergic rhinitis, exercise-induced bronchospasm and food-related flushing and anaphylaxis. A diagnosed of systemic mastocytosis was made and successfully treated with antihistamines, montelukast and cromoglycate. Another case is reported by Sarcina et al. who described an 18-month-old female patient with cutaneous mastocytosis who had severe bullous skin eruption 24 hours after the second dose (booster dose) of inactivated-tetravalent influenza vaccine. The reaction was treated with steroids and antihistamines and further vaccination occurred without adverse events with premedication.

Mast cells have a dual physiological and pathological role, maintaining homeostasis of connective tissues, and being involved in many clinical disorders, particularly hypersensitivity reactions. Animal models of immediate hypersensitivity, as well as *in vitro* and *ex vivo* models of human mast cell activation, in addition to investigation of case series and case reports should lead to a better understanding and management of those conditions.

## Author Contributions

All authors listed have made a substantial, direct, and intellectual contribution to the work and approved it for publication.

## Conflict of Interest

The authors declare that the research was conducted in the absence of any commercial or financial relationships that could be constructed as a potential conflict of interest.

## Publisher’s Note

All claims expressed in this article are solely those of the authors and do not necessarily represent those of their affiliated organizations, or those of the publisher, the editors and the reviewers. Any product that may be evaluated in this article, or claim that may be made by its manufacturer, is not guaranteed or endorsed by the publisher.
